# Proanthocyanidins reduce cellular function in the most globally diagnosed cancers in vitro

**DOI:** 10.7717/peerj.9910

**Published:** 2020-09-15

**Authors:** Sarah Albogami

**Affiliations:** Department of Biotechnology, Faculty of Science, Taif University, Taif, Makkah, Kingdom of Saudi Arabia

**Keywords:** Proanthocyanidins, Anticancer, In vitro, Human colorectal adenocarcinoma, Human breast carcinoma, Human prostatic adenocarcinoma

## Abstract

**Background:**

Growing evidence indicates that proanthocyanidins (PACs) may be effective in treating and preventing various cancers. The fundamental mechanism of PACs inhibiting the proliferation at cellular and molecular levels in most of the cancer types remains unclear.

**Objective:**

The anticancer efficacy of PACs was investigated in vitro using three human cancer cell lines: human colorectal adenocarcinoma (HT-29), human breast carcinoma (MCF-7), and human prostatic adenocarcinoma (PC-3).

**Methods:**

Cytotoxicity was evaluated by MTT assay, while cell proliferation was measured by trypan blue exclusion method. Cell migration was measured by wound healing assay, and DAPI staining was used to evaluate apoptotic nucleus morphology. RT-PCR was used to analyze the expression of *Bax* and *Bcl-2*, and caspase enzyme activity assay was measured by caspase colorimetric assay.

**Results:**

PACs could inhibit both cellular viability and proliferation in a concentration- and time-dependent fashion in all investigated cells. Further, all tested cells showed similarly decreased migration after 24- and 48-h PAC treatment. We observed increased apoptotic nucleus morphology in treated cells (*p* ≤ 0.01). *BAX* expression significantly increased in HT-29 (*p* < 0.01), PC-3(*p* < 0.01), and MCF-7 (*p* < 0.05) cells, while *BCL-2* expression significantly declined (*p* < 0.05). Caspase activities were significantly increased in all tested cancer cell lines after 24-h PAC treatment.

**Conclusion:**

PACs may have potential therapeutic properties against colorectal, breast, and prostate cancer.

## Introduction

Cancer is a major global health problem (Coughlin & Ekwueme, 2009; Jeyaratnam, 1990; Ma & Yu, 2006; Raeisi et al., 2019; Siegel, Miller & Jemal, 2020). Prostate, colorectal, and breast cancers are the most relevant diagnosed cancers worldwide (Chen et al., 2013; Jacobs et al., 2016; Jung et al., 2010; Lee, Oesterreich & Davidson, 2015; Miller & Kolonel, 1996; Parker et al., 1998; Tai et al., 2011). Preventing cancer-related deaths and finding a cure are major research focuses (Gill & Sinicrope, 2005; Stewart, 2012; Thun et al., 2010).

The American Cancer Society has suggested that cancer risks could possibly be prevented by maintaining a healthy diet and lifestyle (Kushi et al., 2006). Fruit and vegetable extracts are a safe, economical source of natural compounds that can be used as chemopreventive agents (Bomser et al., 1996; Nanasombat & Teckchuen, 2009; Wang et al., 2006).

Growing evidence indicates that traditional alternative medicines could be valuable as chemotherapeutic and chemo-preventive agents for drug resistant and malignant tumors (Aggarwal et al., 2006; Dennis, Fanous & Mousa, 2009; Dorai & Aggarwal, 2004; Mehta et al., 2010; Meiyanto, Hermawan & Anindyajati, 2012; Roy, Jauhari & Bharadvaja, 2018; Tan, Gyllenhaal & Soejarto, 2006). PACs are the primary component in grape seeds, and they have antioxidant and anti-inflammatory activities in vitro (González-Quilen et al., 2020; Kaur, Agarwal & Agarwal, 2009; Lei et al., 2020; Lin et al., 2020). Further, PACs influence many genes that are linked to cancer (Kaur, Agarwal & Agarwal, 2009; Ravindranathan et al., 2018; Zhou et al., 2016). The use of PACs in treating and preventing various cancers are the subject of ongoing studies (Chojnacka & Lewandowska, 2020; Pistol et al., 2020; Toden et al., 2018). Researchers have demonstrated that PACs contained in grape seeds are safe for human consumption (Wu, Wang & Simon, 2005).

Grape seed extracts (GSEs) have in vitro cytotoxic effects against several cancer cell lines, while treating normal cells with GSE improves cell viability and growth (Ye et al., 1999). Additional in vitro studies using prostate cancer DU145 cell lines demonstrated that GSEs have anticancer effects via caspase 3-mediated apoptosis and caspase 9 activation (Agarwal, Singh & Agarwal, 2002), or via JNK pathway activation, which inhibits cell growth and induces apoptosis (Tyagi, Agarwal & Agarwal, 2003).

In vivo examinations of GSE effects on animal models of breast cancer demonstrate chemopreventive properties (Kim et al., 2004). Furthermore, GSEs were investigated in a rat dual-organ tumor model (colon and breast tumors), and the results showed that GSEs significantly suppress cancer development (Singletary & Meline, 2001).

GSEs were demonstrated to reduce carcinogenesis via gap junction-mediated cell–cell communication in MCF-7 breast cancer cells. Indeed, the pro-apoptotic properties of GSEs are facilitated by gap junction-mediated cell–cell communication via upregulated connexin *cx43* gene expression (Leone et al., 2019). Furthermore, the use of GSTs in vitro, in the context of HT-29 and LoVo cell cultures and in vivo, in the context of HT-29 tumor xenografts in athymic nude mice revealed that GSTs inhibit cancer cell growth (and volume increase), and upregulate *Cip1/p21* expression (Kaur et al., 2006b).

The fundamental mechanism of inhibition of cell proliferation by PACs at cellular and molecular levels in various cancers remains unclear. Therefore, understanding this mechanism will help to develop and optimize therapeutic approaches to treat several types of cancers.

In this study, the anticancer efficacy of PACs in three human cancer cell lines: human colorectal adenocarcinoma (HT-29), human breast carcinoma (MCF-7), and human prostatic adenocarcinoma (PC-3) cells, which represent the three most commonly diagnosed cancer types worldwide was investigated.

## Materials & Methods

### Cell culture and treatments

**Figure 1:**
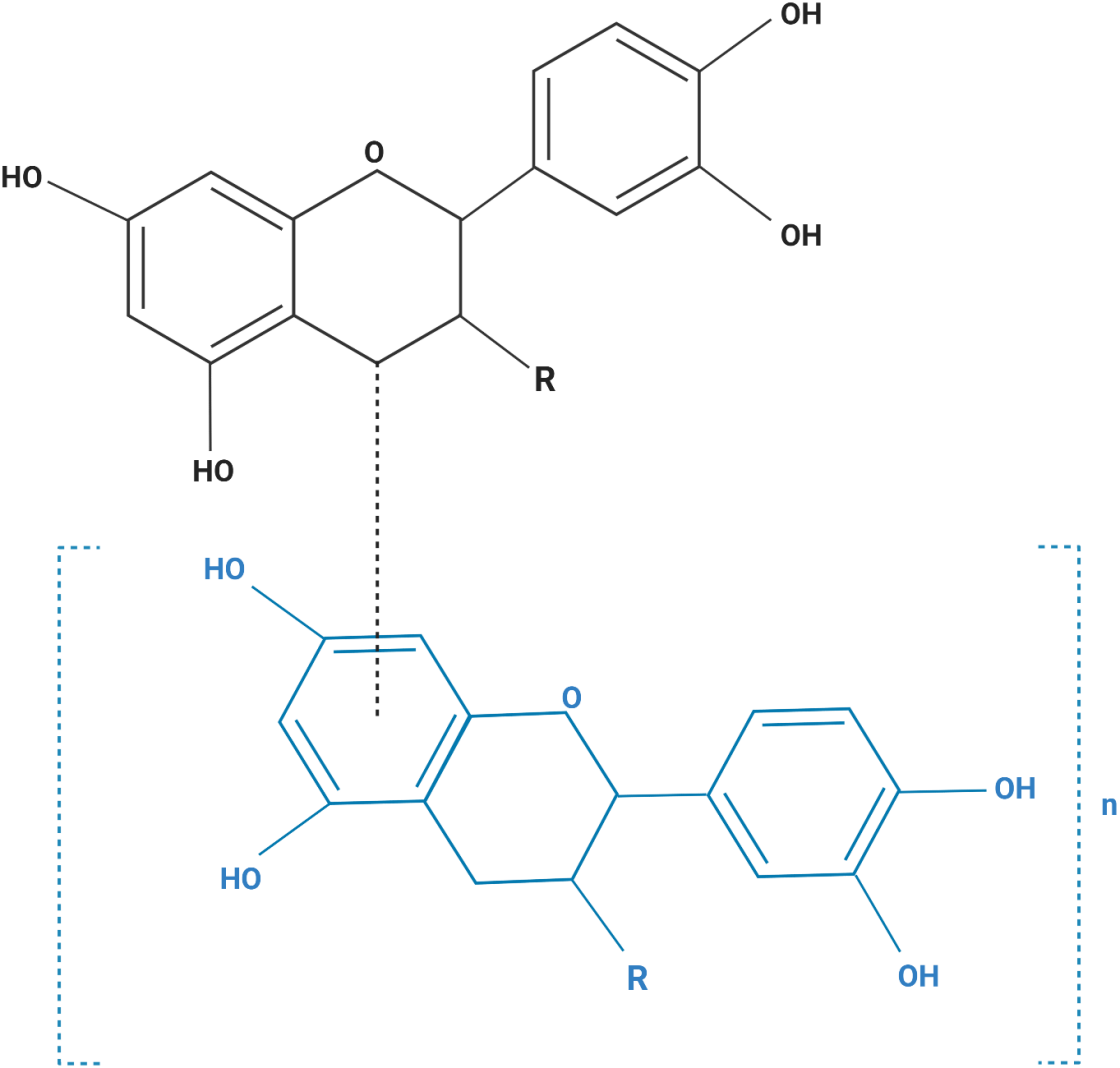
The structural formula of purified grape seed oligomeric proanthocyanidins. Monomer (MW: 290), *n* = 0; dimer (MW: 578), *n* = 1; trimer (MW: 866), *n* = 2; tetramer (MW: 1154), *n* = 3; pentamer (MW: 1442), *n* = 4. R=R3 *β*-OH monomer: catechin (MW: 290); R=R3a-OH monomer: epicatechin (MW: 290); R=R3 *β*-*O*-gallate monomer: catechin 3-*O*- gallate (MW: 442); R=R3a-*O*- gallate monomer: epicatechin 3-*O*- gallate (MW: 442).

The human cell lines used in this study are listed in Table 1. All cells were purchased from the American Type Culture Collection (Manassas, VA, USA). Cells were maintained in media (Table 1) containing 10% fetal bovine serum, 200 mM L-glutamine, 100 U/mL penicillin, and 0.1 mg/mL streptomycin, and maintained at 37 °C in a humidified incubator with 5% CO_2_. Purified grape seeds oligomeric PACs were purchased from Sigma Chemical Co., catalog number (1298219) (St, Louis, MO, USA). Figure 1 shows the structural formula of the PACs obtained. PACs were dissolved in DMSO and diluted with culture media (Khairnar et al., 2018; Liberty et al., 2007; Neto, Amoroso & Liberty, 2008).

**Table 1:** Cancer cell lines used in this study.

Cell Line	Morphology	Origin	Disease	Culture medium
HT29 (ATCC HTB-38)	Epithelial	Colon	Colorectal adenocarcinoma	McCoy’s 5A
PC-3 (ATC CCRL-1435)	Epithelial	Prostate	Grade IV, adenocarcinoma	Roswell Park Memorial Institute RPMI 1640, 10 µg/mL insulin
MCF-7 (ATCC CRL-3435)	Epithelial	Breast	Ductal carcinoma	Minimum essential medium, 10 µg/mL insulin

### Cell viability assay (MTT assay)

Cells were seeded at 1 ×10^4^ cells/well in 96 well flat-bottom plates and cultured for 24 h to allow cells to attach and grow. The next day, the culture medium was exchanged with a serum-free medium to starve cells for 24 h. Then, 0, 5, 10, 50, 100, 200, 400 or 800 µg/mL PACs were added to the cells. The treated cells were incubated for 24 h, 48 h, or 72 h. After each incubation, cells were washed three times with phosphate-buffered saline (PBS). Then, 20 µL 3-[4,5-dimethylthiazol-2-yl]-2,5-diphenyltetrazoliumbromide (MTT, 5 mg/mL, Sigma Chemical Co.) was added to wells and incubated at 37 °C for 3 h. The MTT was removed from each well, and 150 µL DMSO were added and incubated for 5 min at 37 °C. Absorbance was measured at 570 nm (A) using a microplate reader (Spectramax Plus 384, Molecular Devices, USA). The MTT assay was used to determine the IC_50_ value for each cell type at each time point and to evaluate PAC effects on cell viability (Kumar, Nagarajan & Uchil, 2018).

### Cell proliferation

Cells were seeded at 5 × 10^3^ cells/well in 12 well culture plates in complete medium and incubated at 37 °C in a 5% CO_2_ incubator. PACs (0, 5, 10, 50, 100, or 200 µg/mL) were added to each cell type does concentrations, and the treated cells were incubated for 24 h, 48 h, and 72 h. After incubation, trypan blue exclusion was used to count cells. Briefly, 20 µL cell suspension were mixed with 20 µL trypan blue dye 0.4% (Gibco) and incubated at 25 °C for 3 min. 10 µL of the mixture was loaded into a counting slide and counted. Cells were also imaged and counted under bright field magnification after 72-h incubation with 100 µg/mL PACs.

### Wound healing assay

Cells were seeded a density of 20,000 cells/well in 6 well plates and cultured until a monolayer was formed. Then, a straight line was scratched across the center of each well using a sterile 200 µL pipette tip. The culture medium was removed, and each well was rinsed with PBS to remove any cell debris. The cells were treated with PACs at their respective IC_50_ doses. The migration response after scratching and treatment was evaluated. Cells were imaged using an EVOSTM FL cell imaging system (Life Technologies, USA) 0, 24, and 48 h after treatment (Rodriguez, Wu & Guan, 2005). ImageJ software was used to measure the scratch size. Each experiment was performed in triplicate.

### Apoptotic nucleus morphology study

4′, 6′-diamidino-2-phenylindole (DAPI, Life Technologies) was used to observe apoptotic nucleus morphology. Briefly, 2 ×10^5^ cells/well were seeded into 6-well plates and cultured for 24 h. Then, cells were starved for another 24 h. Each cell line was treated with PACs at the respective IC_50_ dose. Then, the cells were rinsed three times with cold PBS and fixed with 100% methanol for 30 min at 25 °C. Cells were stained with 10 µl DAPI (300 nM) for 5 min at 25 °C in the dark. Then, cells were washed several times with cold PBS. Nuclear morphology was observed with an EVOSTM FL cell imaging system (Life Technologies) after 24 h.

### Flow cytometric analysis of apoptosis in HT-29, MCF-7, and PC-3 cancer cells

To evaluate the PACs effects in inducing apoptosis, Annexin V-FITC and propidium iodide (PI) dual staining was used to distinguish apoptotic cells. Annexin V-FITC apoptosis detection kit (BD Bioscience, Franklin Lakes, NJ, USA). In brief, 2 × 10^5^ cells/well were seeded into 6-well plates and cultured for 24 h; then, cells were starved for another 24 h. Each cell line was treated with PACs at the respective IC_50_ concentration for 24 h, while untreated cells were used as control. Culture medium was then discarded, and cells were washed twice with cold PBS and collected, resuspended in a binding buffer. Approximately 100 µl of cell solution was transferred to FACS tube at a concentration of 1 × 10^6^ cells/ml. Approximately 5 µl annexin V-FITC and 5 µl PI were added to each tube and incubated 15 min at 4° C, in the dark; then, 400 µl binding buffer was added to each tube and analyzed immediately using flow cytometer system (FACSCANTO II, BD Biosciences). Weasel v3.6.2 software (Frank Battye, Melbourne, Australia) was used to analyze the results and dot plot the data where cells are described by sections as follows: the live cells are shown in the bottom left section, early apoptotic cells are shown in the bottom right section, necrotic cells are shown in the top left section, and the late apoptotic cells are shown in the top right section.

### RNA isolation

Cells (10^6^ cells/well) were seeded in 6-well plates. Then, cells were treated with PACs as follows: HT-29 cells were treated with 96.62 µg/mL PACs; MCF-7 cells were treated with 115.4 µg/mL PACs; and PC-3 cells were treated with 87.78 µg/mL for 24 h. Untreated cells were used as controls. This PAC concentrations were based on the respective IC_50_ doses determined by the MTT assay. Total RNA from each cell line was isolated with 700 µL TRIzol Reagent (Invitrogen, Carlsbad, CA, USA). A NanoDrop ND-1000 spectrophotometer (Thermo Fisher, Waltham, MA, USA) was used to measure the RNA purity and yield.

### Reverse transcription-polymerase chain reaction (RT-PCR)

An Access RT-PCR System (Promega Corporation, Madison, WI, US) was used to synthesize complementary DNA (cDNA). Target genes were amplified using 1 µM primers specific for *BCL2 associated X* (*Bax)*, *BCL2 apoptosis regulator* (*Bcl-2)*, and *β-actin (ACTB)* (Macrogen Inc, Korea, Table 2). *β-actin* was used to normalized target gene expression.

**Table 2:** Primer sequences, temperatures (Tm), lengths, and product sizes.

**Primer**	**Sequence (5′–3′)**	**Tm** (°C)	**Length** (bp)	Amplicon size (bp)
*Bax* _F	CGAGCAGATCATGAAGACCG	52.9	20	300
*Bax* _R	AAGTAGAACAGGGCCACCAC	53.7	20	
*Bcl-2* _F	AGTACATCCACTACAAGCTGAG	51.4	22	274
*Bcl-2* _R	TACCTCCTGCTGAAGTCGTC	53.0	20	
*ACTB* _F	TCCACGAGACCACCTTCAAC	53.8	20	266
*ACTB* _R	GTACTCCTGCTTGCTGATCC	52.3	20	

Components of RT-PCR mix: 1 X reaction buffer, 0.2 mM dNTP mix, 1 mM MgSO_4_ AMV reverse transcriptase 0.1 u/µL, 0.1 u/µL *Tfl* DNA Polymerase, 1 µg RNA template, and up to 50 µL of nuclease free water. Cycling conditions consisted of two steps: first to synthesis cDNA (1 cycle at 45 °C for 45 min and 1 cycle at 94 °C for 2 min) and second for PCR amplification for 40 cycles of denaturation (94 °C for 30 s), an annealing (52-−53 °C) for 1 min, and an extension step (68 °C) for 2 min. ΔΔCt method was used to calculate the mRNA expression level of both treated cells and control.

### Caspase enzyme activity assay

A Caspase-3, 8, and 9 colorimetric assay kits (GeneTex, Inc., Irvine, CA, USA) were used to evaluate caspase activity. Briefly, 10^6^ cells/well were seeded into 6 well plates and cultured for 24 h. Then, the cells were starved for another 24 h. Cell lines were treated with PACs at their respective IC_50_ doses for 24 and 48 h. Then, cells were harvested and resuspended in 50 µl chilled cell lysis buffer (this buffer is included in the caspase assay kit) and incubated on ice for 10 min. The cells were centrifuged at 10,000*g* for one min and the supernatant was transferred into a new tube. Total protein was measured using a BCA protein assay, and 2 mg/mL BSA was used to generate a standard curve. Approximately 100 µg protein (in the sample supernatant) was diluted in 50 µL cell lysis buffer. Then, 50 µL 2X reaction buffer was added to each sample. Next, 5 µL of 4 mM caspase p-nitroaniline (pNA)-conjugated substrate was added to each sample and incubated at 37 °C for 2 h. Absorbance was measured at 400-405 nm using a microplate reader (Spectramax Plus 384, Molecular Devices).

### Statistical analysis

GraphPad Prism 6 software (La Jolla, CA, USA) was used to perform all statistical analyses. Data are expressed as mean ±  standard deviation (SD) of three biological replicates. The PAC cytotoxicity data in HT-29, MCF-7, and PC-3 cancer cell lines were obtained using non-linear regression analysis. Cellular proliferation and wound healing data were analyzed by two-way ANOVA. Dunnett’s multiple comparisons tests were used to compare the mean of each treatment in each cell line at 0, 24, 48 and 72 h. The wound healing assay data were analyzed using ImageJ software. Apoptotic nucleus morphology, caspase 3 absorbance, and relative gene expression were analyzed using Student’s t-tests. Differences between values were considered statistically significant at *p* ≤ 0.05.

## Results

### PAC cytotoxicity in HT-29, MCF-7, and PC-3 cancer cell lines

**Figure 2:**
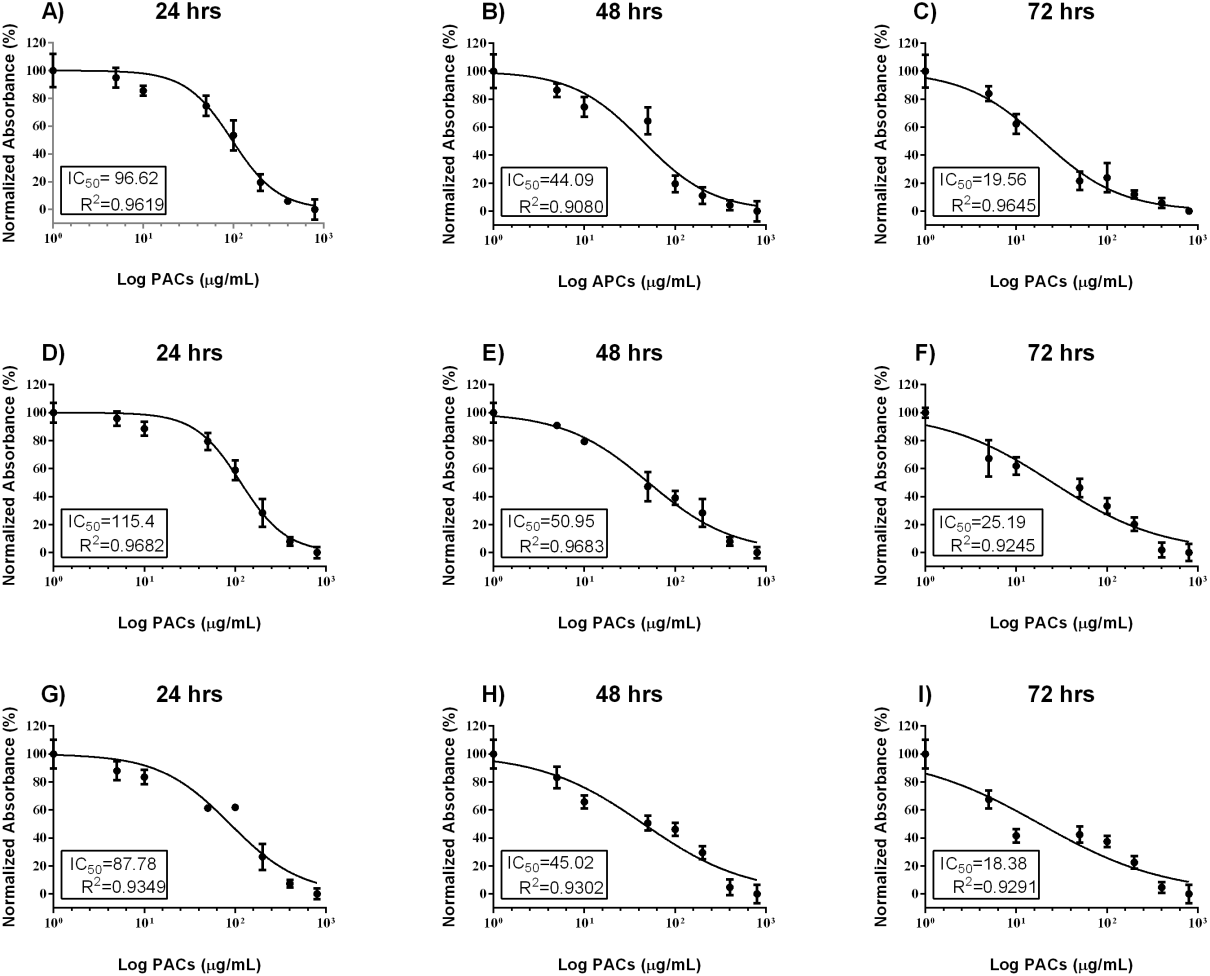
PAC dose-response inhibition curves in HT29, MCF-7, and PC-3 cells. (A–C) HT-29, (D–F) MCF-7, and (G–I) PC-3 cells were exposed to 0, 5, 10, 50, 100, or 200 μ g/mL PACs for 24, 48, and 72 h. Viability was measured by MTT assays. The mean of three experiments (*n* = 3) were plotted to calculate the IC _50_ and R^2^ values by non-linear regression analysis. The error bars represent SD.

**Figure 3:**
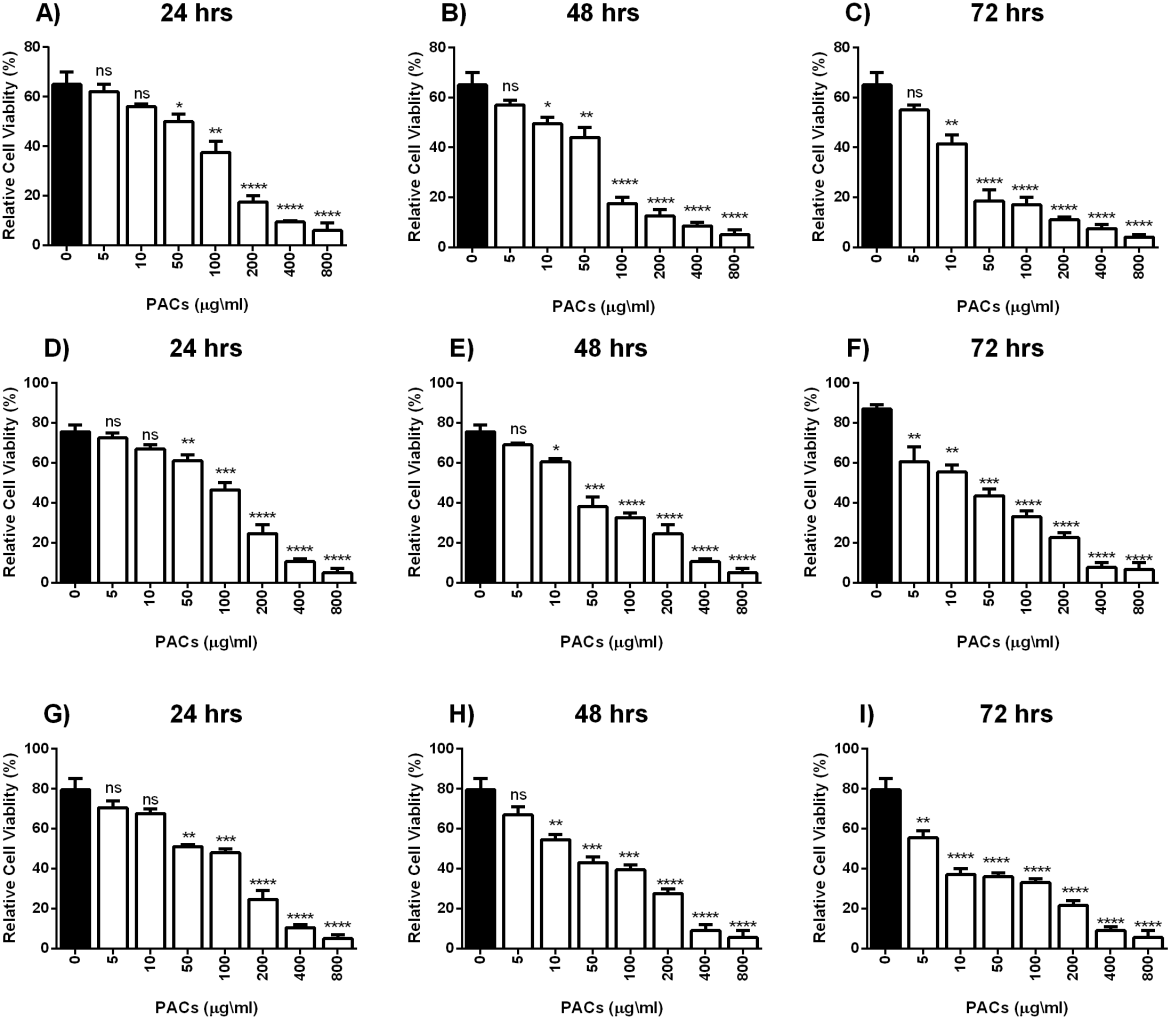
PACs cytotoxicity analysis in HT29, MCF-7, and PC-3 cells. (A–C) HT-29, (D–F) MCF-7, and (G–I) PC-3 cells were exposed to 0, 5, 10, 50, 100, or 200 μ g/mL PACs for 24, 48, and 72 h. Cytotoxicity was measured by MTT assays. The obtained data were analyzed using one-way ANOVA compared to control cells. The data represent the mean ± SD of three independent experiments.

Cell viability was estimated by MTT assays to investigate the cytotoxic effects of PACs on cancer cell lines. HT-29, MCF-7, and PC-3 cells were exposed to 0, 5, 10, 50, 100, 200, 400 or 800 µg/mL PACs for 24, 48 and 72 h. PACs inhibited cellular viability in a dose- and time-dependent fashion in all investigated cells (Fig. 2). The IC_50_ of PACs was calculated to choose the appropriate treatment dose and time for subsequent experiments. PACs showed stronger cytotoxicity against PC-3 cancer cells (IC_50_ = 87.78) than against HT-29 cancer cells (IC_50_ = 96.62) and showed low cytotoxicity against MCF-7 cancer cells (IC_50_ = 115.4). Figure 3 shows that in all three type of cancer cells when treated with PACs for 24, 48 and 72 h there was a major induction of cell cytotoxicity in a concentration- and time-dependent manner.

### PACs inhibit HT-29, MCF-7, and PC-3 cancer cell proliferation

**Figure 4:**
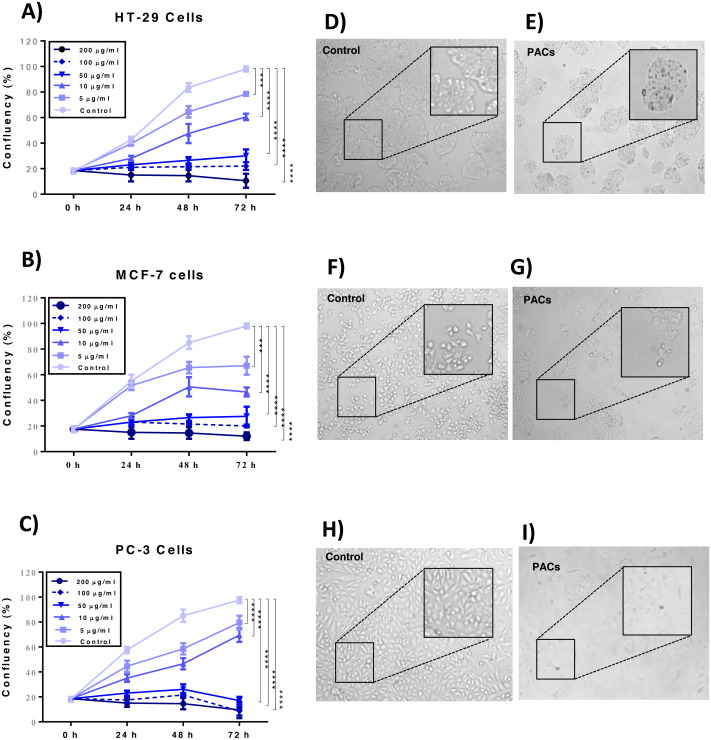
Proliferation of HT29, MCF-7, and PC-3 cells after PACs treatment. (A) HT29, (B) MCF-7, and (C) PC-3 cell confluency percentage compared to untreated cells after 0, 5, 10, 50, 100, or 200 μ g/mL PACs for 24, 48, and 72 h. Cells were counted using trypan blue exclusion. The obtained data were analyzed by two-way ANOVA. The data represent the mean ± SD of three independent experiments. Cell morphology in (D, E) HT29, (F, G) MCF-7, and (H, I) PC-3 cells after treatment with PACs at the LC_50_ dose for 72 h (magnification: 10X). ns: *p* > 0.05, ^∗^*p* < 0.05, ^∗∗^*p* < 0.01, ^∗∗∗^*p* < 0.001 vs. control.

Cellular proliferation was detected by the trypan blue exclusion method to examine the effects of PACs on the cellular proliferation of HT-29, MCF-7, and PC-3 cells. PAC treatment significantly decreased cell proliferation in all investigated cells *p* < 0.001 (Figs. 4A–4C). Further, PACs altered cell morphology after 72 h at their respective IC_50_ doses. We observed that the cells lost typical morphology, becoming fragmented, round, and vacuolated (Figs. 4D–4I). Our results demonstrate that PACs have significant anti-proliferative properties on cancer cell growth.

### Wound healing assay in HT-29, MCF-7, and PC-3 cancer cell lines

**Figure 5:**
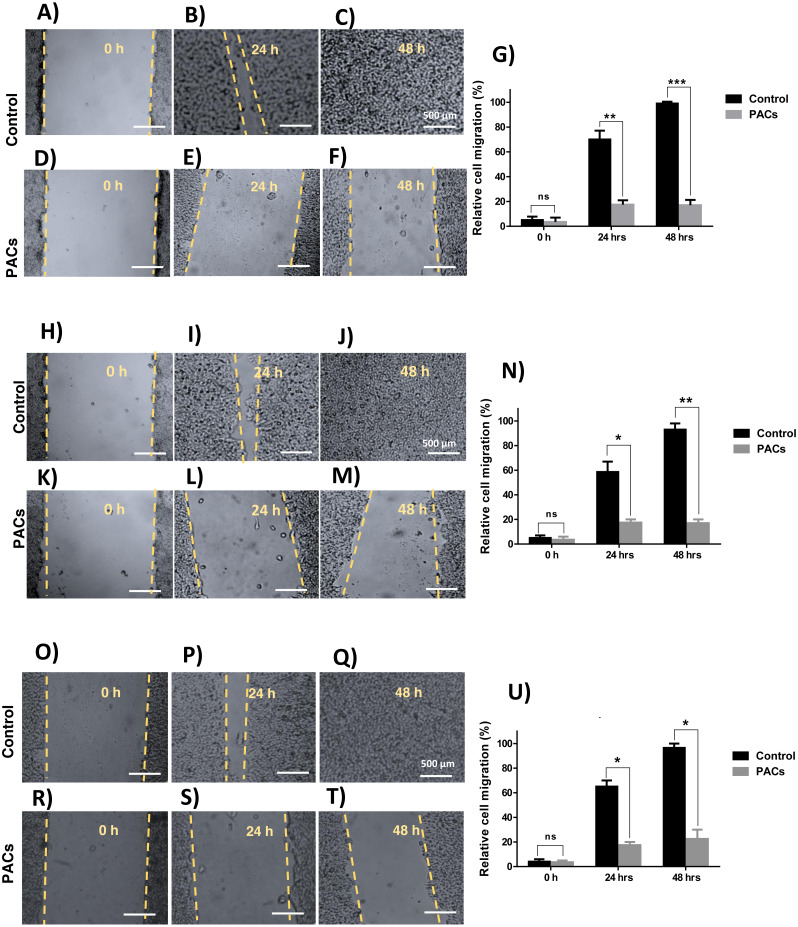
Wound closure in HT29, MCF-7, and PC-3 cells after PACs treatment. In total, 20,000 cells/well were cultured in 6 well plates. After 24 h incubation, a scratch was made in the middle of each well. The cells were treated with PACs at their respective IC _50_ doses. The relative cell migration percentage responses were measured at 0, 24, and 48 h. (A–G) HT29, (H–N) MCF-7, and (O–U) PC-3. The data were analyzed by two-way ANOVA. The mean ±  SD of three biological replicates is plotted, ns: *p* > 0.05, ^∗^*p* < 0.05, ^∗∗^*p* < 0.01, ^∗∗∗^*p* < 0.001 vs. control.

We next investigated wound healing to evaluate cellular migration. Each cell line was treated with 100 µg/mL PACs. All tested cells showed a similarly decreased migration response after 24 and 48 h, indicated by an unhealed wound. Untreated cells showed complete closure of the wound after 48 h (Fig. 5). In all cells, there was no significant cell migration at 0 h. However, untreated cell migration significantly increased after 24 h, compared to HT-29 (^∗∗^*p* < 0.01), MCF-7, and PC-3 cells (^∗^*p* < 0.05). After 48 h, untreated cell migration was significantly increased compared to HT-29 (^∗∗∗^*p* < 0.001), MCF-7 (^∗∗^*p* < 0.01), and PC-3 cells (^∗^*p* < 0.05).

### PACs induce nucleus apoptosis in HT-29, MCF-7, and PC-3 cancer cells

**Figure 6:**
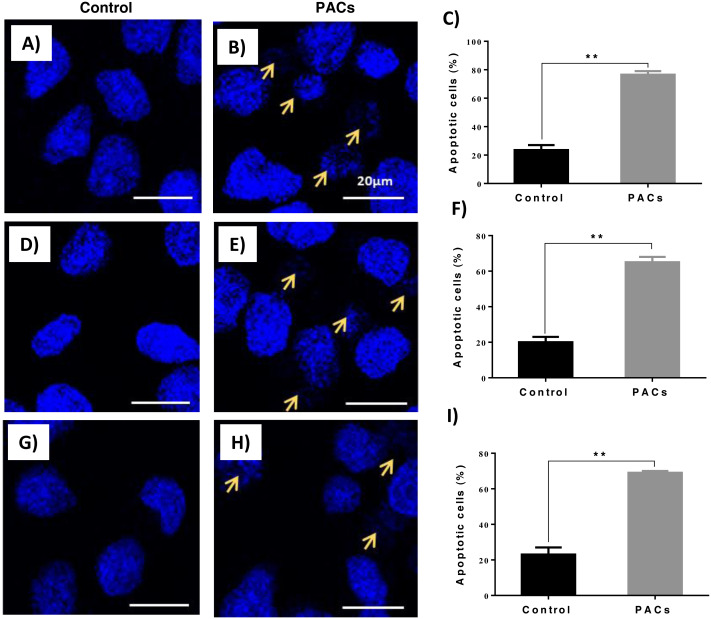
Apoptotic nucleus morphology in HT29, MCF-7, and PC-3 cells after PACs treatment. In total, 2 ×10^5^ cells/well were cultured in 6 well plates. After 24 h incubation, cells were treated with PACs at their respective IC_50_ doses. Nuclear morphology was observed after 24 hours. The obtained data were analyzed using Student’s *t*-test compared to control cells. The mean ±  SD of three biological replicates is plotted. (A–C) HT29, (D–F) MCF-7, and (G–I) PC-3, ns: *p* > 0.05, ^∗^*p* < 0.05, ^∗∗^*p* < 0.01, ^∗∗∗^*p* < 0.001 vs. control.

DAPI staining was used to evaluate apoptotic nucleus morphology in each cell lines after 24 h of PACs at their respective IC_50_ doses. We observed a significant increase in the apoptotic nucleus morphology, including chromatin condensation and apoptotic bodies, in all cell types after PAC treatment (** *p* ≤ 0.01, Fig. 6).

### Flow cytometric analysis of apoptosis in HT-29, MCF-7, and PC-3 cancer cells

**Figure 7:**
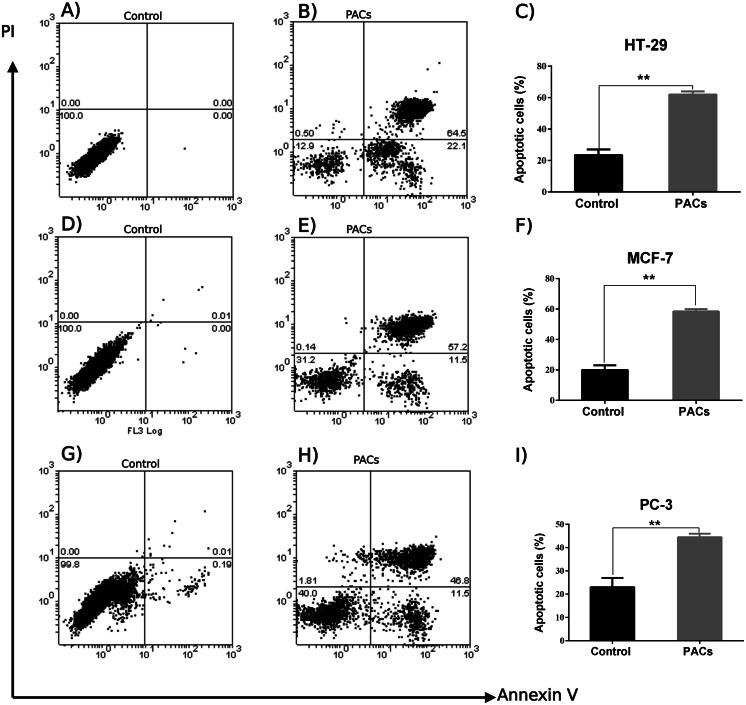
Flow cytometric analysis of apoptosis in HT29, MCF-7, and PC-3 cells following PACs treatment. In total, 2 ×10^5^ cells/well were cultured in 6 well plates. After 24 h incubation, cells were treated with PACs at their respective IC _50_ concentration. After 24 h of treatment, all cells were stained with annexin V-FITC and PI. (A–C) HT29, (D–F) MCF-7, and (G–I) PC-3. The data were analyzed using Student’s *t*-tests compared to control cells. The mean ±SD of three biological replicates is plotted, ns: *p* > 0.05, ^∗^*p* < 0.05, ^∗∗^*p* < 0.01, ^∗∗∗^*p* < 0.001 vs. control.

An annexin V-FITC and PI was used to detect apoptotic cells following PACs treatment in HT-29, MCF-7, and PC-3 cancer cells. The results in Fig. 7 show that the control group (untreated cells) in all three cancer cell lines showed approximately 100% cell viability, while HT-29 cells treated with PACs showed 21.9% viable cells, 0.50% necrotic cells, 22.1% early apoptotic cells, and 64.5% late apoptotic cells (Fig. 7B). MCF- cells treated with PACs showed 31.2% viable cells, 0.14 necrotic cells, 11.5% early apoptotic cells, and 52.2% late apoptotic cells (Fig. 7E), and PC-3 cells treated with PACs showed 40.0% viable cells, 1.81 necrotic cells, 11.5% early apoptotic cells, and 46.8% late apoptotic cells (Fig. 7H). Results shown in Figs. 7C, 7F, 7I demonstrate that in all three cancer cell lines treated with PACs, a significant cell loss (*P* < 0.001) was observed.

### *BCL-2* and *BAX* mRNA expression in HT-29, MCF-7, and PC-3 cancer cells

**Figure 8:**
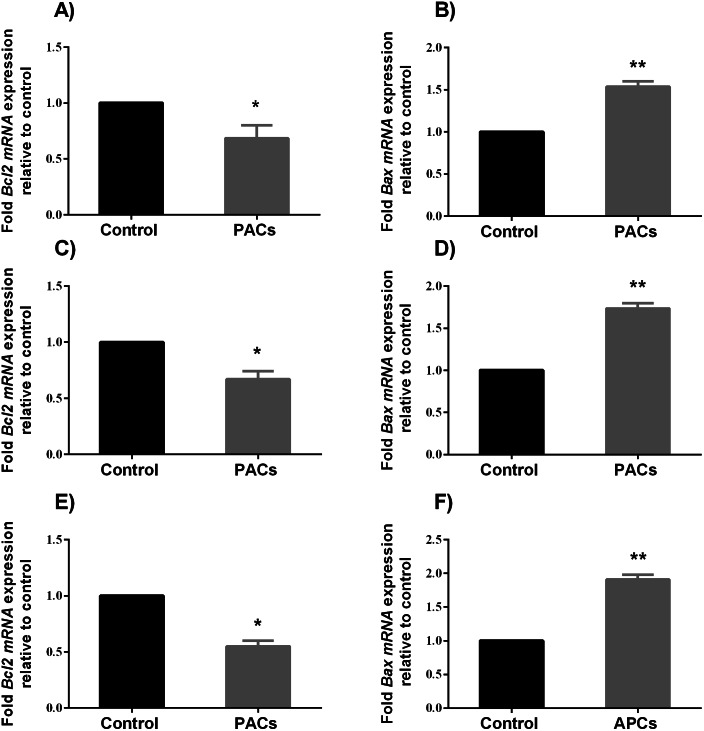
*BCL-2* and *BAX* expression in HT-29, MCF7, and PC3 cancer cells. In total, 10^6^ cells/well were seeded in 6-well plates and treated with PACs at their respective IC_50_ concentration for 24 h. ΔΔ Ct method was used to calculate the mRNA expression level between treated cells and control. The *Y*-axis represents the fold of mRNA expression level. The data were analyzed using Student’s *t*-tests compared to control cells. The mean ±  SD of three biological replicates is plotted. (A, B) HT29, (C, D) MCF-7, and (E, F) PC-3, ns: *p* > 0.05, ^∗^*p* < 0.05, ^∗∗^*p* < 0.01, ^∗∗∗^*p* < 0.001 vs. control.

To determine the ability of PACs to induce apoptosis, we examined the expression of pro-apoptotic genes, such as *BAX*, and anti-apoptotic genes, such as *Bcl-2*, after 24 h exposure to PACs at their respective IC_50_ concentration. *BAX* expression significantly increased (^∗∗^*p* < 0.01 in HT-29 and PC-3 cells and ^∗^*p* < 0.05 for MCF-7 cells), while *BCL-2* expression significantly decreased (^∗^*p* < 0.05, Fig. 8) after treatment with PACs compared to control cells.

### Caspases 3, 8, and 9 enzyme activity assays in HT-29, MCF-7, and PC-3 cancer cell lines

**Figure 9:**
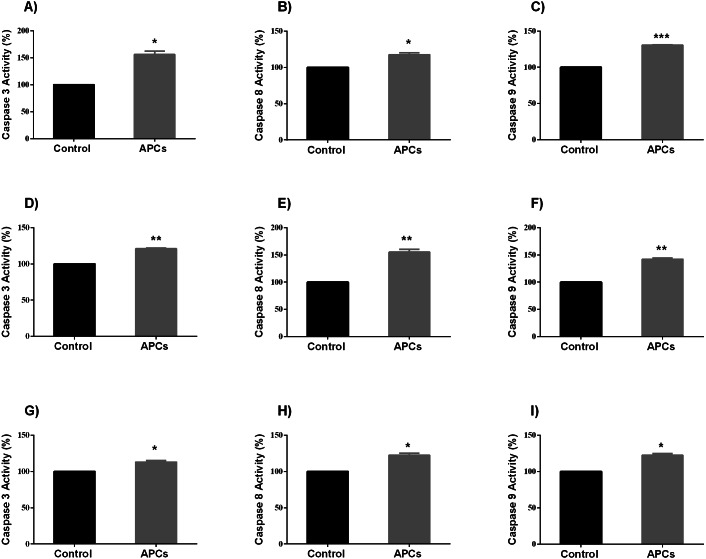
Caspase enzyme activity in HT-29, MCF7, and PC3 cancer cells. In total, 10^6^ cells/well were seeded in 6 well plates and incubated for 24 h. Then, cells were treated with PACs at their respective IC_50_ concentrations for 24 h. The obtained data were analyzed using Student’s *t*-tests compared to control cells. The mean ±  SD of three biological replicates is plotted (A–C) HT29, (D–F) MCF-7, and (G-I) PC-3. ns: *p* > 0.05, ^∗^*p* < 0.05, ^∗∗^*p* < 0.01, ^∗∗∗^*p* < 0.001 vs. control.

It has been shown that the cells undergo apoptosis after caspases activation (Soung et al., 2008). Therefore, in this study the enzymatic activities of caspases 3, 8, and 9 were investigated in all cancer cell lines following PAC exposed for 24 h at their respective IC_50_ concentration. Caspases 3, 8, and 9 enzyme activities were significantly increased. Treated HT-29 cells showed significant increase in caspases activities compared to control (*p* < 0.05, caspases 3 and 8, and *p* < 0.0001, caspase 9; Figs. 9A–9C), while treated MCF-7 cells showed significant increase in caspases 3, 8 and 9 activities compared to control (*p* < 0.01, Figs. 9D–9F); PC-3 cells also showed similar results (*p* < 0.05, Figs. 9G–9I).

## Discussion

Most cancers can be prevented by the maintenance of a healthy diet and lifestyle (Anand et al., 2008). Natural products from plant extracts have the potential to be the next anti-cancer agents (Choi et al., 2017). Several plant-derived molecules (as well as whole plant extracts) have been demonstrated to cure or alleviate several diseases. However, only recently, their potential chemotherapeutic and chemopreventive properties have gained attention (Cragg & Pezzuto, 2016).

In this study, the tumorigenic efficacy of PACs was evaluated in a series of in vitro experiments by using frequently diagnosed human colorectal adenocarcinoma (HT-29), human breast carcinoma (MCF-7), and human prostatic adenocarcinoma (PC-3) cells. These cells were chosen because each cell line represents different cellular signaling and physiological tumor microenvironments.

Our results demonstrate that PACs significantly inhibit HT-29, MCF-7, and PC-3 cell viability in a concentration- and time-dependent fashion. These results corroborate previous studies which assessed the tumorigenic impact of PACs in vitro and showed increased cytotoxicity in colorectal cancer LoVo and HT-29 cells (Kaur et al., 2006a; Leifert & Abeywardena, 2008), Caco-2 cells (Engelbrecht et al., 2007), A-427, A549, and H1299 lung cancer cells (Ye et al., 1999), CRL-1739 human gastric adenocarcinoma cells (Akhtar et al., 2009), Cal27 and SCC25 oral squamous cell carcinoma cells (Chatelain et al., 2011). Importantly, it has been reported that proanthocyanidins (purified from grape seeds) have selective cytotoxicity properties against cancer cells, while they promote the healthy growth of normal cells. In fact, the selective cytotoxicity of PACs has been reported in many human cancer cell lines, like CRL -1739 human adenocarcinoma gastric cell line and A-427 human lung cancer cell line (Ye et al., 1999).

One of the characteristics common to many cancer cells is their ability to proliferate unrestrictedly via apoptosis inhibition (Leone et al., 2019). Importantly, it has been shown that procyanidins from cocoa extracts have the ability to prevent the proliferation of colon cancer cell lines via the blockage of their cell cycle. Furthermore, the anti-proliferative properties of PACs have been reported in murine hepatoma (Hepa1c1c7) cells (Carnésecchi et al., 2002). Herein the obtained results demonstrated that PACs inhibit cellular proliferation in HT-29, MCF-7, and PC-3 cancer cells. Indeed, blocking cancer cell proliferation by inducing apoptosis is a key mechanism of current anti-cancer drugs (Leone et al., 2019). Several morphological alterations can be detected by microscopic examination, especially in the early stage of apoptosis, such as cell shrinkage and DNA fragmentation (Bottone et al., 2013). Our results showed a significant increase in apoptotic features, including chromatin condensation and the formation of apoptotic bodies in all cell types after PAC treatment. Furthermore, flow cytometry analysis showed that PAC treatment results in a decrease in cellular viability and an increase in the percentage of cells in the late apoptotic stage. Once the cell membrane is permeabilized, most of the cells in the early apoptotic stages convert into late apoptotic cells (Aubry et al., 1999). Our results suggest that a small percentage of cells were necrotic, suggesting the involvement of a necrotic cell death pathway. This is not unusual, since, in late apoptosis, cells lose their DNA damage repair capability, leading to cell death and the consequent degradation sometimes in the form of necrotic cells (Krysko et al., 2008). Overall, our results suggest that PACs induce apoptosis in cancer cells.

The potential of PACs to treat resistant and malignant tumors led to the investigation of PAC activity at the molecular level. These studies found that PACs produce reactive oxygen species to stimulate apoptosis pathways (Xu, 2018; Yun et al., 2020). Many pieces of research have proved that the process of apoptosis in cancer cells is usually linked to the overexpression of pro-survival genes, such as some members of the BCL-2 family (divided into two functional subgroups: pro-survival and pro-apoptotic). The *bcl-2* gene encodes a pro-survival factor expressed in both the cell nucleus and cytoplasm. In contrast, the *bax* gene encodes a pro-apoptotic factor, expressed only in the cell cytoplasm. Both BCL-2 and Bax have a fundamental role in the apoptosis regulation process. We speculate that the mechanism behind PACs apoptosis-inducing effects is linked to these two factors. Here, we demonstrated that PACs significantly increase nuclear apoptosis, caspase enzyme activities, and *BAX* gene expression. Additionally, the expression of the anti-apoptotic gene, *bcl-2*, was significantly decreased by treatment with PACs. The overexpression of *bax* (together with the downregulation of *bcl-2*) usually induces the cell to enter the execution stage of apoptosis, leading to the increase in the cleavage of multiple caspases such as caspase-3,-8, and-9 (Del Puerto et al., 2010). In fact, in this study, caspase-3, −8, and -9 enzymatic activities were significantly increased after PACs treatment.

Importantly, the downregulation of anti-apoptotic genes, together with the upregulation of pro-apoptotic genes, is not exclusive to our approach; it is a hallmark of many chemotherapeutic drugs (Huang et al., 2001).

Furthermore, in this study, a wound-healing assay was performed to investigate whether PACs can affect cell motility and migration. Cellular migration and invasion are considered significant features of cancer progression. Importantly, their inhibition promotes better prognostics in vivo (Eccles, Box & Court, 2005). Herein, we demonstrated that all cell lines showed a decrease in cell migration, 24 and 48 h after PACs treatment*.*

**Figure 10:**
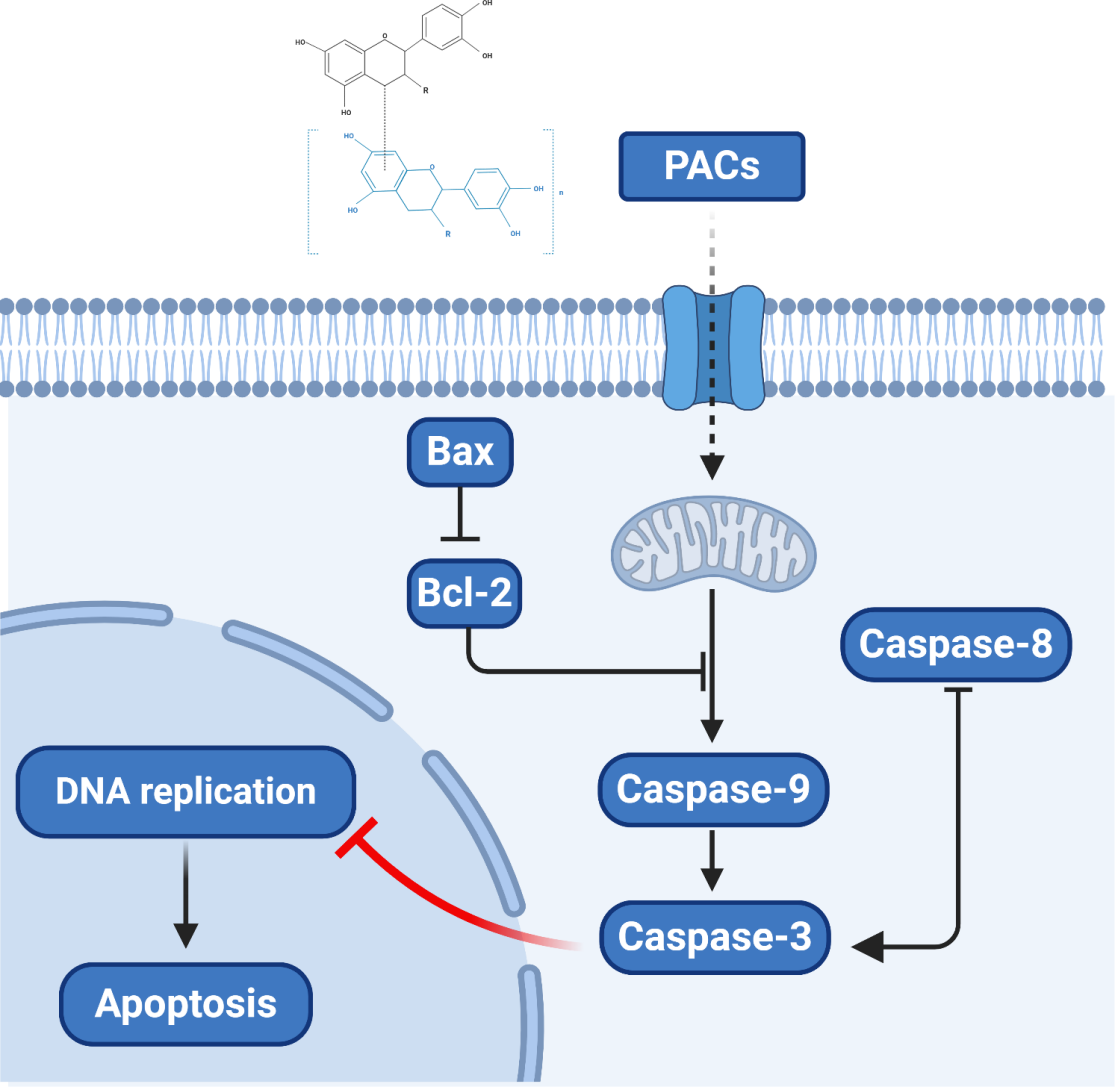
The proposed model of the molecular mechanism underlying the ability PACs to induce cancer cell apoptosis.

Overall, our results provide novel clues regarding the molecular mechanism by which PACs can induce the apoptosis of cancer cells. Proanthocyanidins may exert their anticancer properties via two pathways: the caspase-dependent and the caspase-independent pathways (Clawson et al., 2010; Kim et al., 2010). Based on our observation that PACs activate caspases, downregulate *Bcl-2*, and upregulate *BAX* in cancer cells, we propose a model of the anticancer mechanism of PACs that is summarized in Fig. 10.

The results obtained in this study are, however, preliminary. Further investigations to understand the exact molecular mechanisms behind PACs anticancer effects are needed. Importantly, both in vitro and in vivo contexts should be explored, to fully disclose the role of PACs in the modulation of apoptosis in cancer. Multiple genes layers involved in both extrinsic and intrinsic apoptosis pathways should be explored.

## Conclusions

We showed that PACs inhibit cancer cell viability and reduce proliferation. Further, cancer cell migration was decreased. Additionally, PACs induced nuclear apoptosis, upregulated *Bax* expression, downregulated *Bcl-2* expression was significant, and increased caspase enzyme activity. Thus, PACs demonstrate effective anticancer properties against several cancer types. However, further studies are required in additional cancer types in vitro and in vivo to identify the precise chemopreventive and chemotherapeutic mechanisms of PACs. Further studies using microarrays could be helpful in evaluating the effect of PACs on signaling pathways in cancer cells in comparison with normal cells.

##  Supplemental Information

10.7717/peerj.9910/supp-1 Supplemental Information 1 PACs raw data

## Additional Information and Declarations

### Data Availability

The following information was supplied regarding data availability: The PACs raw data are available as a Supplementary Files.
